# Analysis of variations in cell envelope subproteome and cell length in *Acinetobacter baumannii* ATCC 19606^T^ populations by effect of temperature and desiccation

**DOI:** 10.1007/s10123-025-00706-y

**Published:** 2025-08-23

**Authors:** Maite Orruño, Zaloa Bravo, Iciar Martinez, Inés Arana

**Affiliations:** 1 Department of Immunology, Microbiology and Parasitology, Faculty of Science and Technology, University of the Basque Country UPV/EHU, Barrio Sarriena s/n, 48940, Leioa, Spain; 2 Research Centre for Experimental Marine Biology and Biotechnology (PIE-UPV/EHU), Areatza Hiribidea, 47. 48620, Plentzia, Spain; 3 Department of Zoology and Animal Cell Biology, Faculty of Science and Technology, University of the Basque Country UPV/EHU, Barrio Sarriena s/n. 48940, Leioa, Spain; 4 IKERBASQUE, Basque Foundation for Science, María Díaz de Haro 3. 48013, Bilbao, Spain

**Keywords:** *Acinetobacter baumannii*, Survival strategy, Dryness, Cell envelope subproteome

## Abstract

*Acinetobacter baumannii*, a pathogen for humans and animals, possesses a tremendous potential to survive under hostile conditions. We studied the effect of temperature, nutrient deprivation, and desiccation on the survival of *A. baumannii* ATCC 19606^T^ by monitoring variations in cellular counts and in cell length and analyzing cell envelope subproteome during the survival process. Nutrient deprivation alone does not appear to have a negative effect on *A. baumannii* survival, but incubation at 37 °C in an aqueous solution provoked loss of culturability, as well as a marked increase in cell length. Although a high stability of the membrane subproteome was observed, even under environmental conditions that promote morphological changes and loss of culturability, the expression of some membrane proteins did change upon exposure to the stress. Signal peptidase I and fimbrial protein became undetectable in almost all the conditions examined, while EF-Tu (in all conditions) and MinD (in populations incubated at 37 °C) were overexpressed. The great capacity for survival displayed by *A. baumannii* under adverse conditions may be explained, at least in part, by its capacity to maintain the expression levels of most of its cell envelope proteins and regulate a few others.

**Supplementary Information:**

The online version contains supplementary material available at 10.1007/s10123-025-00706-y.

## Introduction

*Acinetobacter baumannii* is an opportunistic pathogen usually inhabiting the skin, intestinal tract, and respiratory system in mammals, which can cause fatal infections in immunocompromised individuals (Cavallo et al. 2023; van der Kolk et al. 2019; Zou et al. 2023). *A. baumannii* nosocomial infections are especially relevant in intensive clinical and veterinary care unit (ICU) environments (Naing et al. 2022; Nocera et al. 2021).

This pathogen can survive for a long time on inanimate objects such as glass, stainless steel, and other surfaces (Bravo et al. 2019; Espinal et al. 2012; Porter et al. 2024) due to its resistance to desiccation and disinfectants (Bravo et al. 2019; König et al. 2024; Koulenti and Rello 2006) and to its tolerance to starvation and a wide range of temperatures (Bravo et al. 2016; De Silva et al. 2018). *A. baumannii* possesses the ability to remain in the environment and to contaminate surfaces and patients (Islam et al. 2011). Moreover, the main problem related to this pathogen is its ability to develop multidrug resistance and its tendency to spread this resistance to other microorganisms, thus contributing to the development of superbugs (Kyriakidis et al. 2021; Vázquez-López et al. 2020).

Genomics, proteomics, phenotypic analysis, and infection models have been successfully used to screen and identify virulence factors of pathogenic *A. baumannii* (Ayoub Moubareck and Hammoudi Halat 2020), resulting in the identification of capsular polysaccharides, lipopolysaccharides, secretion systems, porins, phospholipase, and iron acquisition systems from cell envelopes as relevant virulence factors (Cavallo et al. 2023; Lee et al. 2018; Morris et al. 2019).

Furthermore, Gram-negative envelopes contribute to key cellular functions, and their composition shows great plasticity and adaptability to environmental variations in order to facilitate survival (Cho et al. 2023; Silhavy et al. 2010). Several studies have determined that the subproteome of the cell envelopes of Gram-negative bacteria undergoes changes under stressful conditions (Orruño et al. 2021; Parada et al. 2016; Schink et al. 2022). Following this line of research, due to the key role of cellular envelopes in bacterial survival and pathogenesis, the aim of the present study was to determine the changes taking place in the protein composition of the cell envelope of *A. baumannii* ATCC 19606^T^ populations under different hostile environmental conditions (starvation, temperature, and desiccation).

## Materials and methods

### Survival assays

*Acinetobacter baumannii* ATCC 19606^T^ was grown overnight at 37 °C in Mueller-Hinton broth with shaking (120 rpm) and transferred to fresh medium up to the stationary phase. The cells were then collected by centrifugation (4500 g, 15 min), washed three times with sterile saline solution (0.9% w/v NaCl [SSS]), and the final pellet was suspended in SSS and used as inoculum for the survival assays.

Survival assays were carried out under nutrient deprivation at two different temperatures (20 °C and 37 °C) in either aqueous or dry conditions as previously described (Bravo et al. 2016, 2019). Nutrient deprivation was performed by incubating the cells in Erlenmeyer flasks containing 1 L of SSS (bacterial density of 10^8^ cells/mL) or on cellulose acetate filters (Whatman [GE Healthcare], Spain) by filtering cellular suspensions through sterile filters (approximately 10^8^ cells/cm^2^) and incubation in sterile Petri dishes. Ambient humidity inside Petri dishes was measured with a Fisher Scientific™ Traceable™ Digital Hygrometer/Thermometer (Thermo Fisher Scientific Inc., Spain) and maintained at a relatively low level (21–27%).

To avoid organic residues, the glass flasks had been previously cleaned with acid, rinsed with deionized water, and kept at 250 °C for 24 h. Filters were sterilized by a 20-min exposure to UV-C (approximately 253.7 nm; 70 mW/cm^2^).

Samples were collected in triplicate periodically along the survival experiments for bacterial enumeration, determination of cell length, and extraction of membrane proteins. Randomly chosen inoculated filters were individually placed in 10 mL of SSS and vigorously shaken for 2 min in order to recover non-planktonic *A. baumannii*.

### Determination of bacterial parameters during the survival assays

The total number of bacteria and cell integrity was determined by the standard acridine orange direct procedure (Hobbie et al. 1977). Culturability was determined by spreading aliquots of *A. baumannii* cells on Mueller-Hinton agar (Scharlab S.L., Spain) followed by incubation at 37 °C for 24 h. The measurements of bacterial length were performed by image analysis of epifluorescence preparations as described by Massana et al. (1997). The image analysis system included a high-resolution video camera (Hamamatsu 2400; Hamamatsu Photonics, Japan). Images of very flat fields with enough bacteria and lacking very bright particles were selected to be digitized and analyzed by Scion Image 1.62ª software. In total, 200 cells were measured in each sample. Three ranges of cell length (I: ≤ x-SD, II: > x-SD - ≤ x+SD, III: > x+SD) were established according to the mean length (x) and standard deviation (SD) of the initial inoculum to estimate the time-dependent changes of cell length in *A. baumannii* populations.

Statistical analyses were carried out with the StatView program (Abacus Concept, Inc.). All the results presented are means of at least three experiments, and the coefficients of variation between replicate experiments were less than 12%. The differences between the means were detected by a one-way analysis of variances. Probabilities less than or equal to 0.05 were considered significant. Logarithmic transformation for bacterial counts was used.

### Isolation of membrane proteins using sodium carbonate extraction

From survival assays carried out at 20 °C and 37 °C, the samples were collected after 30 min (P0), 3 days (P1), and 15 days (P2) of incubation. For survival assays in the aqueous environment, the cells were harvested by centrifugation (8000 g, 40 min, 4 °C), and the pellet obtained was resuspended in 10 mL of Tris-buffered saline pH 7.5 ([TBS] Sigma-Aldrich, Spain). For survival assays on dry surfaces, the samples consisted of five filters resuspended in 10 mL of sterile saline solution and vigorously shaken for 2 min. The cell suspensions were treated as described in previous work (Orruño et al. 2021; Parada et al. 2016). Thus, the cells were harvested by centrifugation (8000 g, 20 min, 4 °C) and resuspended in 10 mL of TBS. Two hundred microliters of Protease Inhibitor Cocktail (Sigma-Aldrich P8465, Spain) per g of cellular weight was added. Then, 90 μL of 2 mM PSMF (Panreac AppliChem, Spain) were added to both types of samples. The suspensions were frozen with liquid nitrogen and stored at −80 °C.

The cells were disrupted by intermittent sonication (SONICS VibraCellTM VCX130 Ultrasonic Cell Disrupter, Sonics, USA) using a 6-mm-diameter probe (65% amplitude setting, 30 s on/45 s off cycles for 3 min total time). Unbroken cells and cellular debris were removed by centrifugation at 6000 g for 20 min at 4 °C. The supernatant fractions were stored on ice, while the pellets were suspended in 10 ml of TBS and sonicated in the same conditions as above. This procedure was repeated at least three times.

The successive supernatants obtained from each sample were combined, diluted (1:1) with 0.2 M sodium carbonate, incubated on ice for 1 h with gentle shaking, and ultracentrifuged at 115,000 g for 1 h at 4 °C. The supernatants were discharged, and protein pellets containing membrane proteins were resuspended in 1 mL of TBS.

### Protein identification and quantification

Analysis of protein samples was performed in the Proteomics Core Facility-SGIKER at the University of the Basque Country, using the protocol previously described by González-Fernández et al. (2013) and used in previous works (Orruño et al. 2021; Parada et al. 2016). Briefly, 50 μg of total protein were precipitated by using a 2-D Clean-Up kit (GE Healthcare, Spain) according to the manufacturer’s instructions. The pellet was suspended in 0.2% RapiGest solution (Waters Corporation, Spain), heated (85 °C, 15 min), reduced with 5 mM DTT, alkylated with 15 mM iodoacetamide, and digested overnight with 2 μg trypsin per sample at 37 °C (Roche Diagnostics, Spain). RapiGest was inactivated by the addition of HCl to a final concentration of 0.5% and incubation at 37 °C for 40 min. Following centrifugation at 16,000 g for 10 min, the supernatant was collected, and MassPREP Enolase Digestion Standard (Waters Corporation, Spain) was added as a standard for protein absolute quantification. Data independent acquisition analyses were performed in a NanoAcquity UPLC System coupled to a SYNAPT HDMS (Waters Corporation, Spain). A final amount of 0.5 μg (containing tryptic peptides and 100 fmol of MassPREP Enolase Digestion Standard) were loaded onto a Symmetry 300 C18, 180 μm × 20 mm precolumn (Waters Corporation, Spain). The precolumn was connected to a BEH130 C18 column (75 μm × 200 mm, 1.7 μm) (Waters Corporation, Spain), and the peptides were eluted with a 120-min linear gradient (3% to 40%) of acetonitrile (v/v) followed by a 15-min linear gradient (40% to 60%) of acetonitrile (v/v). Mass spectra were acquired using a data independent acquisition mode (MSE) described by Silva et al. (2005). Briefly, 1 s alternate MS acquisitions were performed at low (6 eV) and high (12–35 eV ramping) collision energies, and the RF (radio frequency) offset was adjusted so that the MS data were acquired from m/z 350 to 1990. [Glu1]-fibrinopeptide B (Sigma-Aldrich, Spain) at a concentration of 100 fmol/μL was sprayed through the NanoLockSpray source and sampled every 30 s. The obtained spectra were processed with ProteinLynx Global Server v2.4 Build RC7 (Waters Corporation, Spain) using the doubly protonated monoisotopic ion of [Glu1]-fibrinopeptide B for mass correction. Protein identification was carried out by using the embedded database search algorithm of the program (Li et al. 2009) and the UniProtKB database (http://www.uniprot.org/). For protein identification, the following parameters were adopted: carbamidomethylation of C as a fixed modification; N-terminal acetylation, N and Q deamidation, and M oxidation, as variable modifications; 1 missed cleavage and default automatic precursor and fragment error tolerance. A maximum false positive rate of 5% was allowed. Absolute protein quantification was automatically calculated by ProteinLynx Global Server using enolase peptides as a standard (Silva et al. 2006). Only proteins identified with at least three peptides and present in at least two biological replicates were used for absolute quantification. Individual absolute quantification values were normalized using the total protein amount quantified in the sample. Proteins with a significant (*p* < 0.05, t-test) increase or decrease in their relative abundance (>1.5 or <0.66-fold) were considered to be differentially expressed with respect to P0.

UniProtKB and KEGG (http://www.genome.jp/kegg/) databases were used to verify the name, subcellular location, and possible function of the proteins. The subcellular localization of many polypeptides annotated as membrane-associated proteins with known functions was further scrutinized by searching for the cognate membrane-binding domains with the PSORTb v.3.0 program (https://www.psort.org/psortb/). Only polypeptides annotated as proteins with location and/or known function associated with membrane and detected in at least two biological replicates were selected for further analysis. Finally, the selected proteins were sorted according to their main biological functions specified in the UniProtKB database and were further grouped into the following five categories: (i) structural proteins, (ii) in transport, (iii) bioenergetics, (iv) stress response, and (v) miscellaneous functions.

## Results

### Persistence of *A. baumannii* in aqueous or dry conditions

**Fig. 1:**
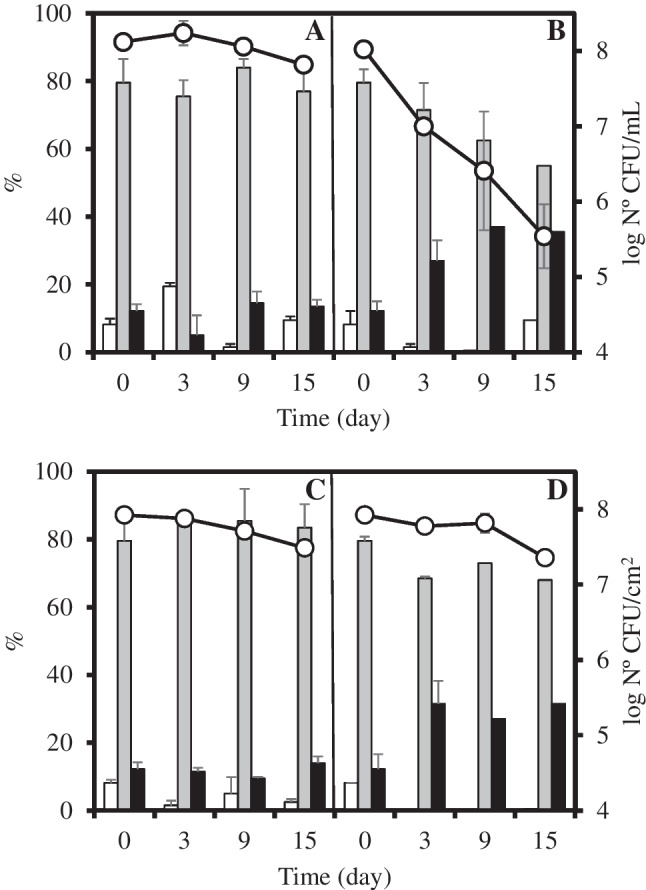
Variations in cell length distributions and CFU counts of *Acinetobacter baumannii* ATCC 19606^T^ populations maintained in aqueous environment (**A**, **B**) and on dry surfaces (**C**, **D**) at 20 °C (**A**, **C**) and 37 °C (**B**, **D**). Cell length distribution: range I (white bar ≤ 0.88 μm), range II (gray bar > 0.88 to ≤ 2.39 μm), and range III (black bar> 2.39 μm). Culturable cells (white circle). The data are mean values from three independent experiments with errors bars representing the standard deviation

The effects of temperature (20 °C or 37 °C) and desiccation on culturability of *A. baumannii* strain ATCC 19606^T^ under nutrient deprivation as well as on the cell size distributions are summarized in Fig. 1.

For the two temperatures studied, microscopic analysis revealed that *A. baumannii* cells, both planktonic and non-planktonic, preserved their integrity throughout the 15 days of experimentation, as indicated by the maintenance of total bacterial counts (data not shown). Moreover, culturability declined slightly but remained nearly unchanged, except for the populations incubated at 37 °C in saline solution (Fig. 1B). In this last case, the culturable cells decreased significantly (*p* ≤ 0.05) already after 3 days of incubation (1.41 ± 0.17 log), and this drop continued after 15 days of experimentation (2.21 ± 0.51 log) (Fig. 1B).

During survival experiments, the cells were assigned to three different groups according to their length (Fig. 1; Table S1). At the beginning of the experiments, the mean cell length was 1.63 (± 0.76) μm, and most of them (> 79%) belonged to range II (> 0.88 to ≤ 2.39 μm). The cell size distribution changed along the survival period, depending on the temperature of incubation. In experiments carried out with populations maintained at 37 °C, significant differences (*p* ≤ 0.05) were detected in the distribution of the length categories. An increase in the number of cells included in range III (> 2.39 μm) took place already after 3 days of incubation at 37 °C, from the initial 12.22%, and it continued to increase in those incubated in saline to around 40% after 6 days (Table S1). Accordingly, the average cell length increased to 2.19 (± 0.55) μm and 2.42 (± 0.59) μm for planktonic and non-planktonic bacterial populations incubated at 37 °C, respectively. In contrast, the cellular length did not vary significantly for the populations maintained at 20 °C.

### Effects of incubation conditions on *A. baumannii* membrane subproteome

Assignment to subcellular locations of the proteins identified in samples collected at different incubation times revealed the presence of proteins from cellular envelopes and cytoplasmic membrane in both planktonic and non-planktonic bacterial populations. Approximately 18% of the identified proteins corresponded to predicted cytosolic proteins; some of them either belonged to cytosolic subunits of membrane protein complexes or were annotated as proteins that can transiently be associated with the membrane (Parada et al. 2016). In all the conditions studied, this percentage increased during the survival period, reaching values greater than 25% of cytoplasmic proteins after 15 days (P3). The highest increase (33%) in the percentage of extracted cytoplasmic proteins was detected during the survival of *A. baumannii* in populations maintained in saline solution at 37 °C.

Proteins that did not undergo a significant variation in their regulation regardless of the experimental conditions are shown in Table 1. The most abundant of them, identified in all the samples, was the porin Omp38 (OMP38_ACIB2), a structural protein associated with *A. baumannii* virulence. Other proteins include proteins involved in maintaining the structure of the cell envelope (D0CBL2_ACIB2 and D0CE28_ACIB2 lipoproteins); related to transport of solutes or protein secretion (D0CDE3_ACIB2, D0CF71_ACIBA2, and D0CF50_ACIB2 porins or YajC subunit); associated with cellular bioenergetics (some ATP synthases, oxidoreductases, cytochromes or succinate dehydrogenase subunits); and related to coping with oxidative stress (catalase, superoxide dismutase, and alkyl hydroperoxide reductase subunit C) or with osmotic stress (D0CDF9_ACIB2) or conferring drug resistance (D0CBN6_ACIB2). The expression of other proteins, such as ATP-dependent zinc metalloprotease FtsH, tyrosine-protein kinase Ptk, or CsuAB, did not change either.

**Table 1:** Membrane proteins of *Acinetobacter baumannii* ATCC 19606^T^ whose level did not change after 3 days (P1) and 15 days (P2) of incubation in saline solution and in polycarbonate filters with respect to initial values (P0)

**Category**	**Protein accession number**	**Location**^**a**^	**Protein name**
Structural	OMP38_ACIB2	OM	Outer membrane protein Omp38
	D0CBL2_ACIB2	OM	Peptidoglycan associated lipoprotein
	D0CE28_ACIB2	CM	NLPA lipoprotein
Transport	D0CDE3_ACIB2	OM	Carbohydrate-selective porin, OprB family
	D0CF71_ACIB2	OM	Carbapenem susceptibility porin CarO
	D0CDN5_ACIB2	OM	Tat pathway signal sequence domain protein
	D0CF50_ACIB2	OM	Putative porin
	D0CDQ0_ACIB2	OM	Outer membrane efflux protein OprM
	D0CD79_ACIB2	CM	Sec translocon accessory complex subunit YajC
	D0C699_ACIB2	CM	Magnesium-transporting ATPase, P-type 1
Bioenergetics	D0CEK6_ACIB2	C	ATP synthase subunit alpha
	D0CEK4_ACIB2	C	ATP synthase subunit beta
	D0CEK8_ACIB2	CM	ATP synthase subunit b
	D0CEK9_ACIB2	CM	ATP synthase subunit c
	D0C5Z4_ACIB2	CM	Cytochrome bo(3) ubiquinol oxidase subunit 1
	D0C6M4_ACIB2	CM	Cytochrome D ubiquinol oxidase, subunit I
	D0C9E0_ACIB2	CM	NADH quinone oxidoreductase subunit A
	D0C9E7_ACIB2	CM	NADH quinone oxidoreductase subunit I
	D0CDS9_ACIB2	CM	Succinate dehydrogenase flavoprotein subunit
	D0CDT0_ACIB2	CM	Succinate dehydrogenase iron-sulfur subunit
Stress	D0C8B2_ACIB2	P	Catalase
	D0CCT4_ACIB2	P	Superoxide dismutase [Cu-Zn]
	D0CBN6_ACIB2	OM	Putative carbapenem-associated resistance protein
	D0CFW7_ACIB2	C	Alkyl hydroperoxide reductase C
	D0CDF9_ACIB2	CM	Large conductance mechanosensitive channel
	D0C9F9_ACIB2	CM	Small-conductance mechanosensitive channel
Others	D0CBC3_ACIB2	CM	ATP dependent zinc metalloprotease FtsH
	D0CEW0_ACIB2	CM	Tyrosine-protein kinase Ptk
	D0C5S9_ACIB2	NC	CsuAB

^a^*OM*, outer membrane; *P*, periplasm; *CM*, cytoplasmic membrane; *C*, cytoplasm; *NC*, non-cytoplasmic proteins according to PSORTb 3.0 program

Changes in the expression of proteins related to the stress conditions are shown in Tables 2 and 3. In most cases, proteins were downregulated. Thus, under starvation, signal peptidase I (D0CBQ1_ACIB2) and fimbrial protein (D0C767_ACIB2) became undetectable. Type VI secretion system effector (Hcp1 family) (D0C8P3_ACIB2) followed a similar pattern but was delayed in the case of planktonic populations (Table 2). Conversely, some proteins were upregulated under stress (Tables 2 and 3), such as elongation factor Tu (D0CG85_ACIB2, EF-Tu) or a protein of the nitroreductase family (D0C6L9_ACIB2), whose expression increased or became detectable throughout the incubation under starvation. Likewise, a temperature of 37 ºC induced the upregulation of the cell division inhibitor MinD protein (D0C9R1_ACIB2) in both planktonic and non-planktonic populations.

**Table 2:** Membrane proteins of *Acinetobacter baumannii* ATCC 19606^T^ present at initial time (P0) and whose level changed after 3 days (P1) and/or 15 days (P2) of incubation in saline solution and/or polycarbonate filters

**Category**	**Protein accession number**	**Location**^a^	**Protein name**	**Saline solution**				**Polycarbonate filters**			
				**20 °C**		**37 °C**		**20 °C**		**37 °C**	
				**P1**	**P2**	**P1**	**P2**	**P1**	**P2**	**P1**	**P2**
Structural	D0CAD0_ACIB2	OM	LPS-assembly lipoprotein LptE	+^b^	+	+	+	+	+	+	-^c^
	D0C9R1_ACIB2	CM	Septum site-determining protein MinD	+	+	+	2.503	+	+	2.363	2.515
	D0C9R5_ACIB2	OM	OmpA family protein	+	0.585^d^	+	+	+	+	+	+
	D0C9S5_ACIB2	OM	Outer membrane protein assembly factor BamE	+	+	+	+	-	-	+	-
	D0CAE4 _ACIB2	OM	Outer membrane protein assembly factor BamB	+	+	+	-	+	+	+	+
	D0C6H3_ACIB2	OM	Outer membrane protein assembly factor BamA	-	-	+	+	+	+	+	+
	D0CEH8_ACIB2	OM	OmpA family protein	-	-	+	+	+	+	+	+
	D0CDK8_ACIB2	C	Cell shape-determining protein MreB	+	+	+	-	-	-	+	+
Transport	D0CDL7_ACIB2	OM	Outer membrane protein transport protein Ompp1/FadL/TodX family	+	2.186	+	+	-	-	+	-
	D0C8P3_ACIB2	NC	Type VI secretion system effector, Hcp1 family	+	0.316	0.200	0.247	-	-	-	-
	D0CDQ2_ACIB2	CM	Acriflavine resistance protein A	-	-	-	-	+	+	+	-
	D0CBQ1_ACIB2	CM	Signal peptidase I	-	-	-	-	-	-	-	-
Bioenergetics	D0C5Z5_ACIB2	CM	Ubiquinol oxidase subunit II	+	+	+	+	+	0.513	+	+
Stress	D0C8Y7_ACIB2	OM	Putative lipoprotein NlpE involved in copper resistance	+	+	+	0.153	+	0.488	+	0,457
Others	D0C9K9_ACIB2	Unknown	LysM domain containing protein	+	+	+	-	-	-	+	+
	D0CCP5_ACIB2	CM	LemA family protein	+	+	+	0.581	+	+	+	+
	D0CCQ1_ACIB2	C	Bacterioferritin	+	+	+	+	0.397	0.485	0.417	0.65
	D0CG85_ACIB2	C	Elongation factor Tu	+	3.296	2.096	2.770	1.550	2.390	1.99	2.02
	D0C767_ACIB2	OM	Fimbrial protein	+	-	-	-	-	-	-	-

^a^*OM*, outer membrane; *P*, periplasm; *CM*, cytoplasmic membrane; *C*, cytoplasmic; *NC*, non-cytoplasmic according to PSORTb 3.0 program. ^b^Value similar to initial. ^c^Undetectable protein. ^d^Values higher than 1.5 indicate significant increases, and values lower than 0.66 indicate significant decreases of protein level with respect to the initial time

**Table 3:** Membrane proteins of *Acinetobacter baumannii* ATCC 19606^T^ undetectable at the initial time (P0) and expressed after 3 days (P1) and/or 15 days (P2) of incubation in saline solution and/or polycarbonate filters

				**Saline solution**				**Polycarbonate filters**			
**Category**	**Protein accession number**	**Location**^a^	**Protein name**	**20 °C**		**37 °C**		**20 °C**		**37 °C**	
				**P1**	**P2**	**P1**	**P2**	**P1**	**P2**	**P1**	**P2**
Transport	D0CFL9_ACIB2	CM	Lipopolysaccharide export system ATP-binding protein LptB	-^b^	-	-	-	-	-	+^c^	+
	D0CE32_ACIB2	P	Curli production assembly/transport component CsgG	-	-	-	+	-	-	-	-
	D0CB10_ACIB2	OM	Outer membrane insertion signal domain protein	-	-	-	+	-	-	-	-
Bioenergetics	D0C9F0_ACIB2	CM	NDH-1 subunit L	-	-	-	+	-	-	-	-
	D0C6L9_ACIB2	Unknown	Nitroreductase family protein	-	+	+	+	-	+	+	+

^a^*OM*, outer membrane; *P*, periplasm; *CM*, cytoplasmic membrane; according to PSORTb 3.0 program. ^b^Undetectable protein. ^c^Detectable protein

The expression of some proteins was clearly dependent on whether the cells were incubated as planktonic or on solid supports, and two differentiated patterns were observed. Some, such as acriflavine resistance protein A (D0CDQ2_ACIB2) became undetectable in planktonic populations, while others, such as bacterioferritin and D0CDL7_ACIB2 transport protein, became downregulated or undetectable in non-planktonic populations regardless of incubation temperature. The combination of stressors affected differentially the expression of other proteins. For instance, BamA (D0C6H3_ACIB2) and an OmpA family protein (D0C9R5_ACIB2) were downregulated in saline solution at 20 °C, while LemA family protein (D0CCP5_ACIB2) experienced a similar change in planktonic populations but at 37 °C, and putative lipoprotein NlpE involved in copper resistance (D0C8Y7_ACIB2) decreased under all conditions except when maintained at 20 °C in saline solution. Other proteins, such as MreB (D0CDK8_ACIB2) followed different patterns depending on the combination substrate-temperature: it was detectable during the entire period at 20 °C but not after 15 days at 37 ºC; but on filters, it was detectable only at 37 °C.

Finally, some proteins, undetectable at P0, such as Curli production assembly transport component CsgG (D0CE32_ACIB2), outer membrane insertion signal domain protein (D0CB10_ACIB2) and NDH-1 subunit L (D0C9F0_ACIB2), became detectable after 15 days of permanence in saline solution at 37 °C (Table 3). Likewise, lipopolysaccharide ABC transporter LptB (D0CFL9_ACIB2) became detectable in populations maintained on solid surfaces at 20 °C.

## Discussion

*A. baumannii* is an opportunistic pathogen that causes serious nosocomial infections largely due to its ability to resist stressful conditions and spread in the hospital environment (Naing et al. 2022; Zeidler and Müller 2019). In the present study, the survival patterns of *A. baumannii* ATCC 19606^T^ populations, maintained under adverse conditions (nutrient deprivation, non-optimal temperature, and/or relative desiccation), showed a similar evolution of total and culturable populations to that previously described (Bravo et al. 2016, 2019). Nutrient deprivation alone does not appear to have a negative effect on the survival up to 15 days at 20 °C, but incubation at 37 °C in aqueous medium provoked loss of culturability, as well as a marked increase in cell length (Fig. 1).

There seemed to be a discordance between the apparent loss of cell culturability in planktonic populations at 37 °C and the manifestation of some activities. Thus, after 3 days of incubation, the percentage of cells longer than 2.39 μm increased by approximately 15%, and the variation in the proteome of cellular envelopes (Table 2) and the identification of proteins not detectable in the inoculum, such as CsgG, were observed (Table 3).

This work also analyzed how the environment affects the proteins present in the cell envelopes. It is noteworthy that a significant percentage of predicted cytosolic proteins was detected, presumably due to contamination (Parada et al. 2016; Zhu et al. 2022). In addition, nutrient deprivation increased the levels of these cytosolic proteins (from 18% to values greater than 25%), especially in the conditions that had the greatest deleterious effect on the culturability of *A. baumannii* (37 °C in saline solution), when 33% of detected sequences corresponded to cytoplasmic proteins. Therefore, there seems to be an association between both parameters, stress and increased contamination of samples with cytosolic proteins.

In the present work, even under environmental conditions that promote loss of culturability and morphological changes, high stability of membrane subproteome was observed (Table 1). Thus, the maintenance of several structural proteins (such as lipoproteins or Omp38) can be correlated with the maintenance of the integrity of the cell envelopes (data not shown) and, therefore, of cell structure (Lin et al. 2002). Likewise, all the proteins identified related to bioenergetics kept or increased their expression levels under given conditions, even in mainly non-culturable populations. The trend was similar for proteins involved in transport; only a few displayed decreased expression or became undetected during the survival period (Table 2).

Consistent detection, during at least 15 days of exposure to adverse environmental conditions, of catalase, superoxide dismutase, and alkyl hydroperoxide reductase (Table 1), could indicate that the cells are able to cope with a stable level of oxidative stress for prolonged periods of time. Moreover, the consistent detection of the large conductance mechanosensitive channel D0CDF9_ACIB2 could prevent disruptions in cellular osmotic pressure (Booth and Blount 2012). In this context, it is also remarkable the stability in the detection of the ATP-dependent zinc metalloprotease FtsH, which degrades some misassembled membrane proteins, thus contributing to the maintenance of its correct functionality (Narberhaus et al. 2009; Yang et al. 2019).

Several authors have demonstrated changes in the proteome of Gram-negative bacteria, including *Acinetobacter* spp., maintained under adverse conditions (Gayoso et al. 2014; Guo and Gross 2014; Parada et al. 2016). The present work corroborates those results demonstrating that responses to stress caused by fluctuations in environmental conditions induced changes in the expression of certain membrane proteins. Apparently, *A. baumannii* limits the expression of proteins that are not essential for survival, as signal peptidase I and fimbrial protein, while maintains the levels of those necessary for adaptation to environmental stress. In this regard, the disappearance of fimbrial protein, involved in biofilm formation (Choudhary et al. 2022), would support the decreased ability to form biofilms previously detected (Bravo et al. 2016) and indicate that this ability could not be necessary for survival. On the other hand, the non-detection of signal peptidase I, together with the fact that hardly any increase in the expression of membrane proteins was detected, may indicate that the turnover of membrane proteins decreased substantially. In support of this, Bravo et al. (2016) and König et al. (2024) detected a significant decrease in the expression of *csuAB* gene in populations maintained under adverse conditions; nevertheless, in the present work, the levels of CsuAB protein remained constant, indicating that degradation of some previously synthesized proteins may not occur. This could be the situation for other proteins relevant for survival as well, such as those related to transport, bioenergetics, or responses to stress.

Remarkably, the presence of EF-Tu in cell envelopes increased under stress conditions, even though it is mainly a cytoplasmic protein involved in protein translation. This protein has been localized extracellularly in many bacterial pathogens, where it is involved in several cellular processes, including stress responses and pathogenesis (Harvey et al. 2019; Wang and Jeffery 2016). Furthermore, its presence in membrane is compatible with the disappearance of signal peptidases, since transport of cytosolic proteins to cell surface is independent of signal sequences and can occur through multiple mechanisms (Harvey et al. 2019). Indeed, EF-Tu has been previously detected in membrane vesicles derived from *A. baumannii* (Kwon et al. 2009).

Bacteria can alter their morphology in response to environmental conditions (Justice et al. 2014; Yang et al. 2016). Thus, several authors have indicated that the persistence of many Gram-negative bacteria under adverse environmental conditions is accompanied by a progressive reduction of cell length and changing cell morphology from rod to coccoid shape (Krebs and Taylor 2011; Orruño et al. 2021; Sun et al. 2016). This response, mainly driven by starvation, is accelerated by other abiotic stresses including temperature, visible light, and salinity (Orruño et al. 2021; Parada et al. 2016). Adoption of a coccoid shape would allow cells to minimize the resources necessary to maintain cell viability and to enhance nutrient uptake under limitation of nutrients (Parada et al. 2016). In contrast, other authors have shown filamentation to be the most frequent shape change due to limitation in the availability of one or more nutrients and could be definitely important during the infectious cycle of pathogenic bacteria (Young 2006) and exert a role in subverting host innate defenses (Justice et al. 2014). In concordance with the latter authors, the present work showed that elongation was the main trend for both planktonic and non-planktonic populations incubated at 37 °C, which is the temperature in human hosts.

We also identified two proteins potentially involved in this morphological change, namely the actin homologue MreB and a component of the MinCDE system, both belonging to independent bacterial cytoskeletal-like systems (Shih et al. 2005). The MreB protein, required for the maintenance of a rod-shaped appearance (Govindarajan and Amster-Choder 2016), showed random behavior that was unrelated to the variations in the size distribution of the populations. Evidence suggesting the transient association of this protein with the membrane could explain the absence of a pattern in its detection (Laddomada et al. 2016). However, incubation at 37 °C induced an overexpression of the cell division inhibitor MinD in both planktonic and attached populations. The components of the MinCDE system, together with the nucleoid, regulate the localization of the FtsZ protein, which is involved in leading septum formation and determines cellular shape (Wu et al. 2015). Overexpression of MinD causes the formation of filamentous forms in *Bacillus subtilis* (Bramkamp and van Baarle 2009) and prevents division in *Escherichia coli* (Howard et al. 2001), even though these results may be consistent with the increase in enlarged cells for populations incubated at 37 °C in this study.

Finally, it is important to note that while findings using the ATCC 19606^T^ strain provide useful foundational knowledge, caution is necessary when extrapolating these results to clinical isolates of *A. baumannii*. Clinical strains exhibit considerable genetic and phenotypic heterogeneity, resulting in strain-dependent differences in stress adaptation, survival strategies, and phenotypic traits relevant to clinical outcomes (Bravo et al. 2019; Valcek et al. 2023). Consequently, the behaviors observed in this study may not fully represent the diversity present in clinical populations.

In summary, *A. baumannii* ATCC 19606^T^ shows a great capacity for survival under conditions of nutrient deprivation, maintaining integrity and culturability and, largely, cell length. The exception is found in planktonic populations maintained at 37 °C: under these conditions, loss of culturability and a remarkable enlargement of their cell size were noted, changes that have been related to the infectious cycle in other pathogenic bacteria. Regarding cell envelope proteins, *A. baumannii* ATCC 19606^T^ seems able to maintain most of its proteins under stress conditions, which would explain its high persistence under adverse conditions. Nevertheless, the turnover rate of these proteins is apparently low throughout survival.

## Supplementary Information

Below is the link to the electronic supplementary material.

Supplementary file1 (DOCX 17 KB)

## Data Availability

No datasets were generated or analysed during the current study.
