# Supplementary material for: Analysis of variations in cell envelope subproteome and cell length in Acinetobacter baumannii ATCC 19606T populations by effect of temperature and desiccation
Source: Int Microbiol. 2025 Aug 23;28(8):2539–49. doi: 10.1007/s10123-025-00706-y (PMC12727854; doi:10.1007/s10123-025-00706-y)
Supplement: Supplementary file 1 — Supplementary file1 (DOCX 17 KB) [file 10123_2025_706_MOESM1_ESM.docx]

**Table S1** Variations in mean cell length (μm) and length range distribution (%) in *Acinetobacter baumannii* ATCC 19606^T^ populations maintained, at 20ºC and 37ºC, in saline solutions (planktonic cells) and on polycarbonate filters (non-planktonic cells). Cell length distribution is presented as percentage of cells in range I, ≤ 0.88 μm; range II, > 0.88 - ≤ 2.39 μm and range III, > 2.39 μm.

|  | **Saline solution** | | | | | | | | **Polycarbonate filters** | | | | | | | |
| --- | --- | --- | --- | --- | --- | --- | --- | --- | --- | --- | --- | --- | --- | --- | --- | --- |
|  | **20ºC** | | | | **37ºC** | | | | **20ºC** | | | | **37ºC** | | | |
| **Day** | **Length** | **Ranges** | | | **Length** | **Ranges** | | | **Length** | **Ranges** | | | **Length** | **Ranges** | | |
|  |  | **I** | **II** | **III** |  | **I** | **II** | **III** |  | **I** | **II** | **III** |  | **I** | **II** | **III** |
| **0** | 1.63  (0.76)^a^ | 8.17  (0.91) | 79.61  (6.91) | 12.22  (2.02) | 1.63  (0.76) | 8.17  (0.91) | 79.61  (6.91) | 12.22  (2.02) | 1.63  (0.46) | 8.17  (0.91) | 79.61  (6.91) | 12.22  (2.02) | 1.63  (0.76) | 8.17  (0.91) | 79.61  (6.91) | 12.22  (2.02) |
| **3** | 1.31 (0.76) | 19.51  (1.17) | 75.50  (4.80) | 4.99  (0.59) | 2.07 (0.41) | 1.62  (1.01) | 71.31  (3.94) | 27.07  (2.82) | 1.93 (0.32) | 1.55  (0.41) | 77.40  (6.37) | 21.05  (1.98) | 2.22 (1.04) | ND^b^ | 68.53  (6.24) | 31.43  (4.44) |
| **6** | 1.84 (0.84) | 10.45  (0.61) | 79.05  (6.17) | 10.50  (0.74) | 2.38 (0.42) | 0.20  (2.01) | 59.20  (5.11) | 40.60  (3.10) | 1.76 (0.87) | ND | 83.50  (10.51) | 16.50  (1.75) | 2.05 (0.85) | 0.52  (0.43) | 79.31  (7.52) | 20.17  (3.24) |
| **9** | 1.70 (0.64) | 1.51  (1.62) | 83.79  (7.02) | 14.70  (3.56) | 2.21 (0.39) | 0.50  (0.50) | 62.49  (2.33) | 37.01  (3.92) | 1.52 (0.81) | 5.00  (2.12) | 85.50  (7.83) | 9.50  (2.42) | 2.14 (0.73) | ND | 73.03  (5.46) | 26.97  (1.25) |
| **12** | 1.77 (0.85) | 5.67  (1.28) | 76.55  (6.01) | 17.78  (3.34) | 2.29 (0.63) | 5.52  (1.15) | 60.77  (4.32) | 33.71  (3.25) | 1.59 (0.71) | 1.56  (0.25) | 87.94  (6.62) | 10.50  (2.63) | 2.07 (0.76) | ND | 74.65  (4.82) | 25.45  (3.34) |
| **15** | 1.58 (0.79) | 9.52  (1.01) | 77.00  (5.61) | 13.48  (3.40) | 2.19 (0.55) | 9.62  (4.05) | 54.82  (8.10) | 35.56  (6.21) | 1.69 (0.62) | 2.32  (1.82) | 83.46  (9.41) | 14.22  (3.41) | 2.42 (0.59) | 0.50  (0.04) | 68.05  (5.53) | 31.45  (6.82) |

a, Standard Deviation. b, No Detected.
